# Placenta: an old organ with new functions

**DOI:** 10.3389/fimmu.2024.1385762

**Published:** 2024-04-19

**Authors:** Sara Khorami-Sarvestani, Negar Vanaki, Sorour Shojaeian, Kayhan Zarnani, Allan Stensballe, Mahmood Jeddi-Tehrani, Amir-Hassan Zarnani

**Affiliations:** ^1^ Reproductive Immunology Research Center, Avicenna Research Institute, ACECR, Tehran, Iran; ^2^ Monoclonal Antibody Research Center, Avicenna Research Institute, ACECR, Tehran, Iran; ^3^ Department of Immunology, School of Public Health, Tehran University of Medical Sciences, Tehran, Iran; ^4^ Department of Biochemistry, School of Medical Sciences, Alborz University of Medical Sciences, Karaj, Iran; ^5^ Department of Health Science and Technology, Aalborg University, Aalborg, Denmark; ^6^ Clinical Cancer Research Center, Aalborg University Hospital, Aalborg, Denmark

**Keywords:** placenta, proteins, pregnancy, immunomodulation, embryo implantation, cancer, biomarker

## Abstract

The transition from oviparity to viviparity and the establishment of feto-maternal communications introduced the placenta as the major anatomical site to provide nutrients, gases, and hormones to the developing fetus. The placenta has endocrine functions, orchestrates maternal adaptations to pregnancy at different periods of pregnancy, and acts as a selective barrier to minimize exposure of developing fetus to xenobiotics, pathogens, and parasites. Despite the fact that this ancient organ is central for establishment of a normal pregnancy in eutherians, the placenta remains one of the least studied organs. The first step of pregnancy, embryo implantation, is finely regulated by the trophoectoderm, the precursor of all trophoblast cells. There is a bidirectional communication between placenta and endometrium leading to decidualization, a critical step for maintenance of pregnancy. There are three-direction interactions between the placenta, maternal immune cells, and the endometrium for adaptation of endometrial immune system to the allogeneic fetus. While 65% of all systemically expressed human proteins have been found in the placenta tissues, it expresses numerous placenta-specific proteins, whose expression are dramatically changed in gestational diseases and could serve as biomarkers for early detection of gestational diseases. Surprisingly, placentation and carcinogenesis exhibit numerous shared features in metabolism and cell behavior, proteins and molecular signatures, signaling pathways, and tissue microenvironment, which proposes the concept of “cancer as ectopic trophoblastic cells”. By extensive researches in this novel field, a handful of cancer biomarkers has been discovered. This review paper, which has been inspired in part by our extensive experiences during the past couple of years, highlights new aspects of placental functions with emphasis on its immunomodulatory role in establishment of a successful pregnancy and on a potential link between placentation and carcinogenesis.

## Introduction

“Yet, in spite of being the star of the show, the placenta has never quite managed to gain the attention it deserves. Compared to other organs of the body, the placenta comes very low down the list when measured on the scale of public awareness. It is not the placenta’s fault that it has languished in such obscurity. No one has bothered to speak up on its behalf and put it firmly into the public domain.” (1)

- Y. W. Loke

## Placental evolution in mammals

The way in which mammals reproduce is the major focus of evolutionary biology, and understanding these processes underpins much of the diversity in our lives. After many years of evolution from the egg-laying ancestor of vertebrates, viviparity converges and involves numerous anatomical, behavioral, genetic, and physiological changes to support internally-incubated embryos. The transition from oviparity to viviparity and the establishment of feto-maternal communications to provide nutrients, gases, and hormones to the developing fetuses introduced the ‘placenta’ as the anatomical mediator of exchange between fetal membranes. The time and approaches that viviparous mammals, marsupials, and eutherians invest in birth differ from one another. The brain size of the neonates in each species is considered one factor that may influence the type of maternal investment (1).

Egg-laying monotremes like the Platypus and Echidna lack a placenta and other reproductive compartments such as the uterus; however, marsupials like kangaroos, koalas, bandicoots, and opossums have a functional placenta. The gestation time in marsupials is as short as 12 days, so much of maternal investment focuses on lactation for the better development of the little joeys. It has been shown that the mammary glands of marsupials function as an alternative to the eutherian placenta, and the composition of milk gradually changes as the offspring grows (2). Delving into the transcriptome changes in the placenta of a small Australian marsupial, the tammar wallaby, researchers have found that marsupials have a eutherian-like placenta with similarly differentially expressed genes as the eutherian placenta in the early stages of pregnancy (3). This suggests that placental functions are the same in the two species, and the difference might lie in the way of compartmentalization and the type of maternal investment in each species.

**Figure 1:**
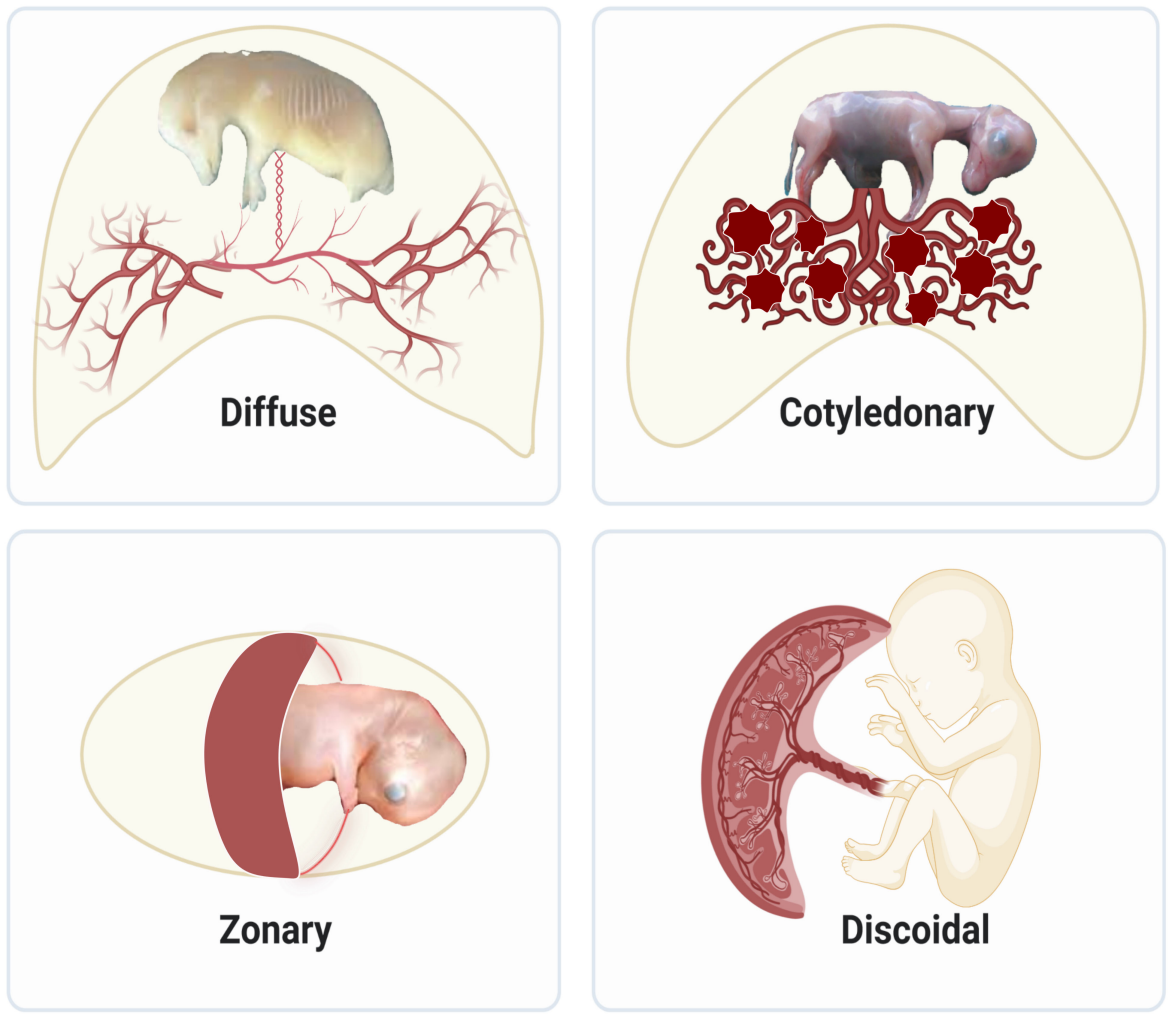
Structural diversity of placentas in mammals: Diffuse placentas have a uniform distribution of chorionic villi covering the chorion’s surface and are seen in horses and pigs. Cotyledonary placentas have numerous button-like structures called cotyledon that join together using vascularized intervening areas to form placentome. This type of structure is seen in sheep, goat, and cattle. Zonary placentas are like a belt created around the chorionic sac and are seen in carnivores like dogs, cats, and seals, omnivores like bears, and herbivores like elephants. Discoidal placentas form disc-like structures and this placental structure is seen in rodents and primates like humans.

Much of our knowledge on placenta development comes from animals in the eutherian lineage, which comprises over 5000 vertebrates? species and 20 phylogenetic orders. The different sizes, gross shapes, and histological structures of layers that separate maternal and fetal circulations result in various types of placentas. Regarding size and shape, placentas are classified as diffuse, cotyledonary, zonary, and discoid/bidiscoid types (**Figure 1**). In the diffuse type, as seen in horses and pigs, the entire surface of the allantochorion is involved in the formation of the placenta. The interaction of multiple patches of allantochorion with the endometrium results in cotyledonary placenta, observed in ruminants. Zonary is a complete or incomplete band of tissue that surrounds the fetus, found in carnivores. The last type is discoid/bidiscoid, confined to a roughly circular area and seen in primates, rodents, and rabbits (4).

**Figure 2:**
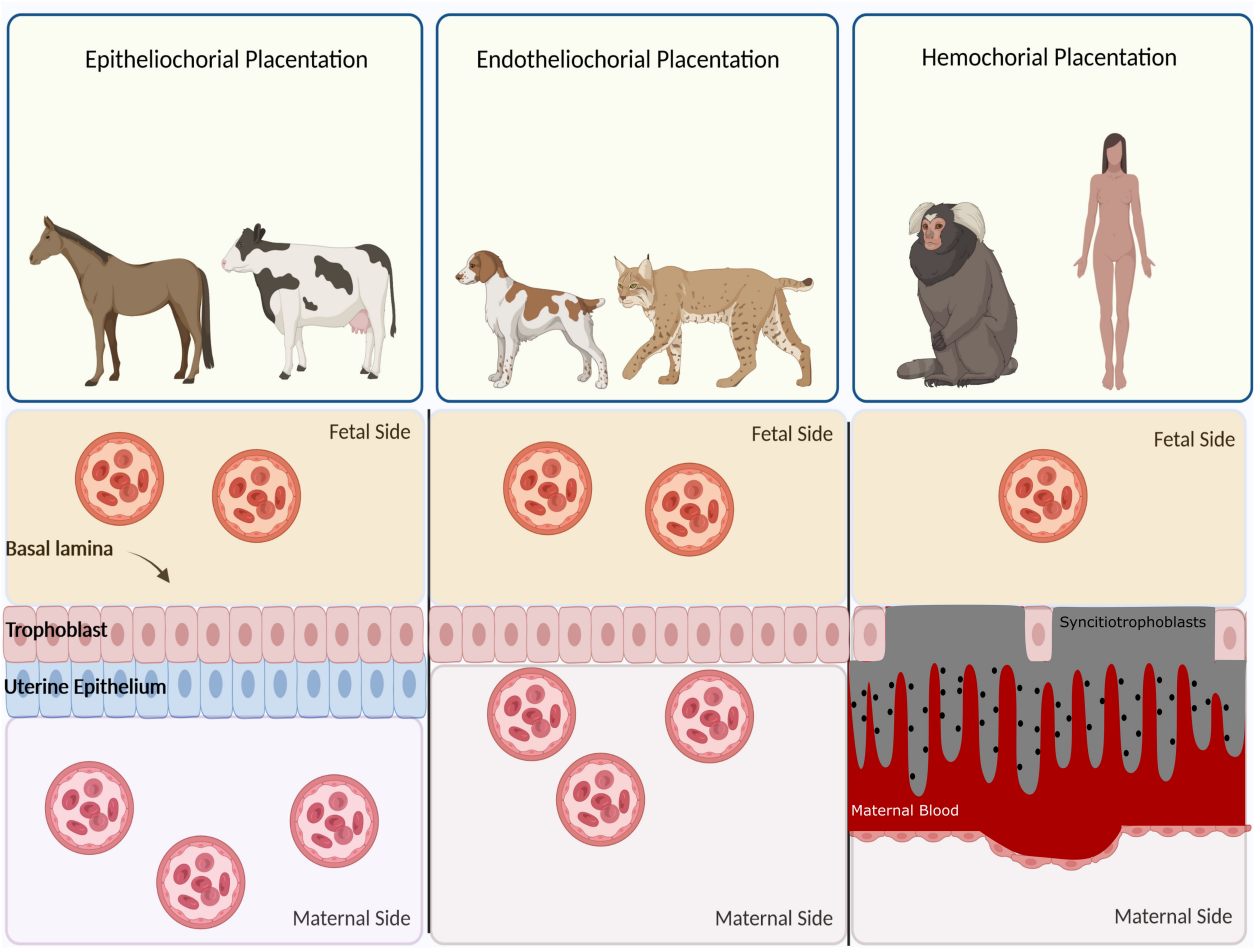
Classification of placentas based on cell layers comprising the maternal-fetal interface. In epitheliochorial placentation, as seen in cow, horse, and ruminants, the least intimate interactions occur between fetal tissue and maternal blood since a barrier containing maternal vascular endothelium and uterine epithelium exists. In endotheliochorial placentation, as seen in dogs and cats, trophoblasts invade the uterine epithelium and contact with maternal endothelium. In hemochorial placentation, as seen in primates and humans, the most intimate contact occurs between fetal trophoblasts and maternal circulation since both the epithelium and endothelium degrade and trophoblasts are soaked in the maternal blood.

Based on histological structure, four types of placentation have emerged, namely epitheliochorial, endotheliochorial, haemochorial, and synepitheliochorial (**Figure 2**). In species with non-invasive epitheliochorial placentation, such as horses and pigs, pockets of columnar trophoblasts loosely attach to the endometrial epithelium. Endotheliochorial placentation involves a medium degree of invasion, seen in carnivores. The most invasive type is haemochorial, as seen in humans, rats, and mice, in which the trophoblast is in direct contact with maternal blood. In synepitheliochorial placentation, trophoblast cells fuse with uterine epithelial cells to form syncytium. This type is observed in ruminants; however, some developmental biologists believe it has evolved from haemochorial placentation and named it secondary epitheliochorial placentation (5). The change in the type of placentation in ruminants might result from a positive pleiotropic effect, as described first for the relationship between placentation and cancer malignancy (see below for more information).

Some researchers posit that marsupials possess a non-invasive placenta; however, recent hypotheses have indicated that the placenta of marsupials is also invasive. The challenge lies in the brief gestation period, which precludes the observation of changes related to placental invasiveness. This limitation has been compensated for by a relatively longer lactation period to better nourish the joeys (6).

In addition to the gestational period, maternal homeostatic mechanisms that cope with embryo-induced inflammation also determine the degree of invasiveness. This means that the short period of fetus implantation in marsupials is followed by parturition. In contrast, in eutherians, severe inflammation associated with deep placental invasion is compensated for by anti-inflammatory mechanisms (7). Therefore, deep placental invasion occurs in species that have evolved mechanisms to handle severe inflammation. Among the most important compensatory mechanisms is the platelet/megakaryocyte system responsible for hemostasis. The degree of invasion is much more related to maternal balancing systems than the nature of the placenta itself. Hemostatic mechanisms are needed to repair the destruction of blood vessels upon hemochorial placentation in eutherians (8). The evolution of the megakaryocyte platelet system emerged before the evolution of deep placentation. This suggests that deep invasion occurs in species that have learned how to deal with undesired outcomes. This idea echoes the words of the evolutionary biologist Theodosius Dobzhansky: ‘Nothing in biology makes sense except in the light of evolution.’”

## An overview on placental development in humans and rodents

While human and mouse placentas share similar overall structures and molecular mechanisms underlying development, there are notable differences in their gross structures. Assuming the formation of the vaginal plug as day 0.5 of mouse pregnancy (9, 10), placental development begins at 3.5 days post conception (dpc) when the inner cell mass and outer trophoectoderm layers are established (10, 11). At the time of implantation (4.5 dpc), trophoblasts, corresponding to the area opposite and farthest from the inner cell mass, differentiate into polyploid trophoblast giant cells analogous to human extravillous cytotrophoblast cells (EVT) (12). Trophoectoderm cells adjacent to the inner cell mass, polar trophoectoderm, differentiate into the two main diploid cell types: the extraembryonic ectoderm and the ectoplacental cone (11).

In later stages of mouse placental development, the extraembryonic ectoderm forms the chorion layer and later the labyrinth. The ectoplacental cone differentiates into the spongiotrophoblasts, which form a compact layer between the labyrinth and the outer giant cell layer and is analogous to the column cytotrophoblast of the human placenta (10, 12). Subsequently, glycogen trophoblast cells are formed within the spongiotrophoblast layer (11). The allantois, formed at the posterior end of the developing embryo (8 dpc), is the origin of the vascular portion of the placenta (11).

The fusion of chorionic mesothelium and allantois occurs at 8.5 dpc, followed by the folding of the chorionic mesothelium, creating spaces into which fetal blood vessels grow from the allantois (11). This event coincides with the differentiation of chorionic trophoblast cells into two labyrinth cell types. In a process quite similar to human placental development, trophoblast cells of the labyrinth layer adjacent to the fetal endothelium fuse with each other, forming multinucleated syncytiotrophoblast cells (STB). The second cell type differentiated from labyrinth trophoblast cells is a mononuclear trophoblast cell that lines the maternal blood sinuses. Fetal vessels and labyrinth trophoblasts generate branched villi, analogous to human chorionic villi, which continue to grow and branch until 18.5-19.5 dpc. Notably, maternal and fetal blood circulate in opposite directions, maximizing nutrient transport (11).

Human placental development begins 6-7 days after conception, around the attachment of the blastocyst to the uterine wall (13). In humans, the polar trophoectoderm adheres to the endometrial epithelium, while in mice, it is the mural trophoectoderm that contacts the endometrium (14). Once attached, the polar trophoectoderm rapidly proliferates and forms a regional bilayer trophoblast, consisting of an inner layer of rapidly proliferating cytotrophoblasts (CTB) and an outer layer of multinucleated STB, differentiated by CTB fusion (15). Although STB are non-proliferative in essence, they can combine with the CTB basal cell layer and continue to proliferate throughout pregnancy (16). Trophoblast invasion into the maternal decidua is an essential step for the initiation of subsequent fetal development, including vasculature remodeling and crosstalk with endometrial decidual stromal cells (DSC) and immune cells residing in the decidua (17).

The development of human placental villi starts around 8 dpc through the proliferation of CTB and folding of the outer primitive STB layer into the decidua. The first villus mesenchymal cores are formed by the invasion of extraembryonic mesenchyme into the villi at around 12 dpc Endothelial cells can be identified within the villus core from 15 dpc. Similar to mouse villi, endothelial cords within human placenta villi are in close contact with the trophoblast basement membrane, implying that trophoblast cells may contribute to villous vascular network development through a paracrine manner. At around 18-20 dpc, the first fetal capillaries and placental macrophages (Hofbauer cells) appear in the stroma of placental villi. The development of placental villi capillaries continues, and at around 22 dpc, branching vessels are developed by connecting endothelial cell cords to each other and elongating parallel to the villus axis. The villi blood vessels connect to the fetal circulation via the umbilical cord around 32 dpc (18).

Villi structure in humans and mice is structurally similar, surrounded by a trophoblastic bilayer consisting of mononuclear trophoblasts and an overlying multinucleated STB layer. STB is in close contact with maternal blood and is the major site for the absorption of nutrients and oxygen and waste exchange. STB actively involves the production of human chorionic gonadotropin (hCG) and progesterone, necessary for fetal development. Around 15 dpc, CTB at the tips of anchoring villi rapidly proliferate, detach from villi columns, and differentiate into EVT (19). Two types of EVT can be identified: interstitial CTB (iCTB), which is highly invasive and invade decidual stroma, playing a fundamental role in shaping endometrial immune regulation (see below). Endovascular CTBs (eCTB) invade uterine spiral artery lumens and replace endothelial cells to ensure adequate placental perfusion as pregnancy proceeds. eCTB form trophoblastic plugs during the first weeks of pregnancy until around 7 weeks of gestation and prevent blood flow in the spiral arteries. This event provides a hypoxic microenvironment and is thought to play a major role in placental development (18). In the second and third trimesters of pregnancy, placental villi continue to mature, characterized by a densely packed stroma. In parallel, placental villi vasculature undergoes extensive expansion through branching angiogenesis. In the third trimester, vascular branching stops, and capillary loops are formed, further increasing exchange between maternal and fetal circulations.

## The role of placenta in maintenance of pregnancy

The placenta performs numerous vital biological functions, including nutrient and waste transfer, gas exchange, and the transmission of maternal immunoglobulins to the fetus, providing passive immunity (20). Additionally, it contributes to fetal development through hormone secretion (21). The proximity of the placenta and decidual blood arteries is carefully regulated to meet the developing fetus’s higher metabolic needs by adjusting the endometrial circulation appropriately (22). It’s noteworthy that the human placenta possesses safeguards preventing the passage of potentially harmful compounds. Export pumps in the STB’s maternal-facing membrane, along with the expression of cytochrome P450 (CYP) genes involved in xenobiotic metabolism during the first trimester of pregnancy, contribute to these protective characteristics (23, 24).

The placenta also acts as a safeguard against the vertical transmission of infectious agents. While few pathogens can infect the placenta and fetus in humans, maternal infection doesn’t guarantee placental or fetal infection (25). The placenta employs physical and immunological mechanisms to resist infection. The STB layer, consisting of almost 60 billion nuclei, lacks cell junctions, forming a barrier against microbial attachment and invasion. The microvasculature of fetal blood vessels restricts access of microorganisms to fetal blood. The maternal decidua, rich in leukocytes, serves as an effective first-line immunological defense. Various cells, such as decidual natural killer (dNK) cells and DSCs, contribute to placental innate immune defenses. The maternal decidua is a leukocyte-rich layer and constitutes an effective first-line immunological defense at the maternal–fetal interface. As an example, dNK cells could transfer granulysin to trophoblasts and confer resistance without actively killing these cells. DSCs, as the most frequent decidual cells, have a major role in placental innate immune defenses. Villous trophoblast, in addition to providing a physical defense, also acts as a chemical barrier to the vertical transmission of microorganisms. For instance, human trophoblasts secrete antiviral interferons (type III) that restrict infection in both an autocrine and a paracrine manner. Interestingly, placental extracellular vesicles (EV) contained antiviral microRNAs with broad antiviral activity. Inflammasome-associated cytokines such as Interleukin (IL)-1β, IL-18, and IL-1α have been reported to protect placental cell infection with L. monocytogenes (25). As an example of an effective function of the placenta against infection, it is noteworthy that only 1.5-2% of pregnancies in HIV-positive women appear to result in the vertical transmission of HIV across the placenta (26).

Additionally, the placenta is a source of autocrine, paracrine, and endocrine factors. Placental hormones can be broadly categorized into those acting at the feto-maternal interface, mediating communication between the mother and developing fetus, and those exerting regulatory effects in remote targets in maternal compartments. These regulatory effects are essential for maternal physiological adaptations during pregnancy, such as changes in maternal circulation, development of lactating glands, provision of hemotroph to the embryo, and parturition. Sex hormones, including estrogen and progesterone, play a crucial role in various functions such as endometrial differentiation, myometrial quiescence, cervical closure, local immunotolerance in the pregnant uterus (27–29), trophoblast differentiation, autoregulation of placental steroidogenesis, regulation of the maternal cardiovascular system, uteroplacental blood flow, placental neovascularization and mammary gland development (30). Growth hormone (GH) and prolactin (PRL) are well-studied placental hormones with significant activity in mediating maternal metabolic adaptations to pregnancy. Placental hormones also impact maternal neuroendocrine organs, including the brain, hypothalamus, and pituitary glands, facilitating maternal adaptation to pregnancy and maintaining homeostasis. Neuroactive hormones like melatonin, serotonin, and oxytocin are involved in these activities. Other placental hormones, such as activins, relaxin, leptin, and parathyroid hormone-related protein (PTHrP), play roles in mediating changes in maternal vascular function and metabolism (26). The immunoregulatory role of leptin in the human placenta has been discussed further in the next sections.

## Placenta function in decidualization and implantation

In species with hemochorial placentation, the placenta must invade the endometrial spiral arteries to create sufficient blood supply for nourishing the developing embryo. This invasion, completed at the end of the first trimester, is regulated by the local decidual environment (31). According to an evolutionary hypothesis, known as the precondition hypothesis, the uterus undergoes various cellular and molecular alterations to be adapted for potential pregnancy. One mechanism for such adaptations is the extensive structural and molecular changes in endometrial stromal cells under the control of ovarian steroid hormones, collectively referred to as decidualization (32, 33). Decidualization occurs in the mid-to-late secretory phase of the menstrual cycle in menstruating species; however, in non-menstruating species, it occurs only after implantation. The decidual environment consists of DSCs, immune cells, and extracellular matrix (34).

Decidualization serves to protect the endometrium from oxidative stress and hyper-inflammation during the deep invasion of the placenta in hemochorial placentation (32, 34). In humans, decidualization coincides with the recruitment of uterine natural killer cells (uNK) to the uterus via local expression of chemokines such as CCL4, CXCL9, and CXCL10. This cell type is a rich source of growth and angiogenic factors and is the most frequent immune cells of the uterus that modulate T cell functions through the expression of glycodelin-A and galectin-1 (33). The endometrial immune system plays a fundamental role in immune-based uterine preconditioning necessary for blastocyst implantation. The activation of redox-sensitive signaling pathways, resistance to cell death, and the expression of angiogenic factors such as vascular endothelial cell growth factor (VEGF) are among the most important mechanisms for the immune-based uterine preconditioning process (32).

Decidualization has both protective and supportive roles in placental development. It provides cytokines and growth factors necessary for remodeling the implantation site, while simultaneously limiting the degree of placental invasion to prevent complications such as placenta accreta. The depth of trophoblast invasion in mammals, which differs according to the type of placentation, correlates with the extent of decidualization (35).

**Figure 3:**
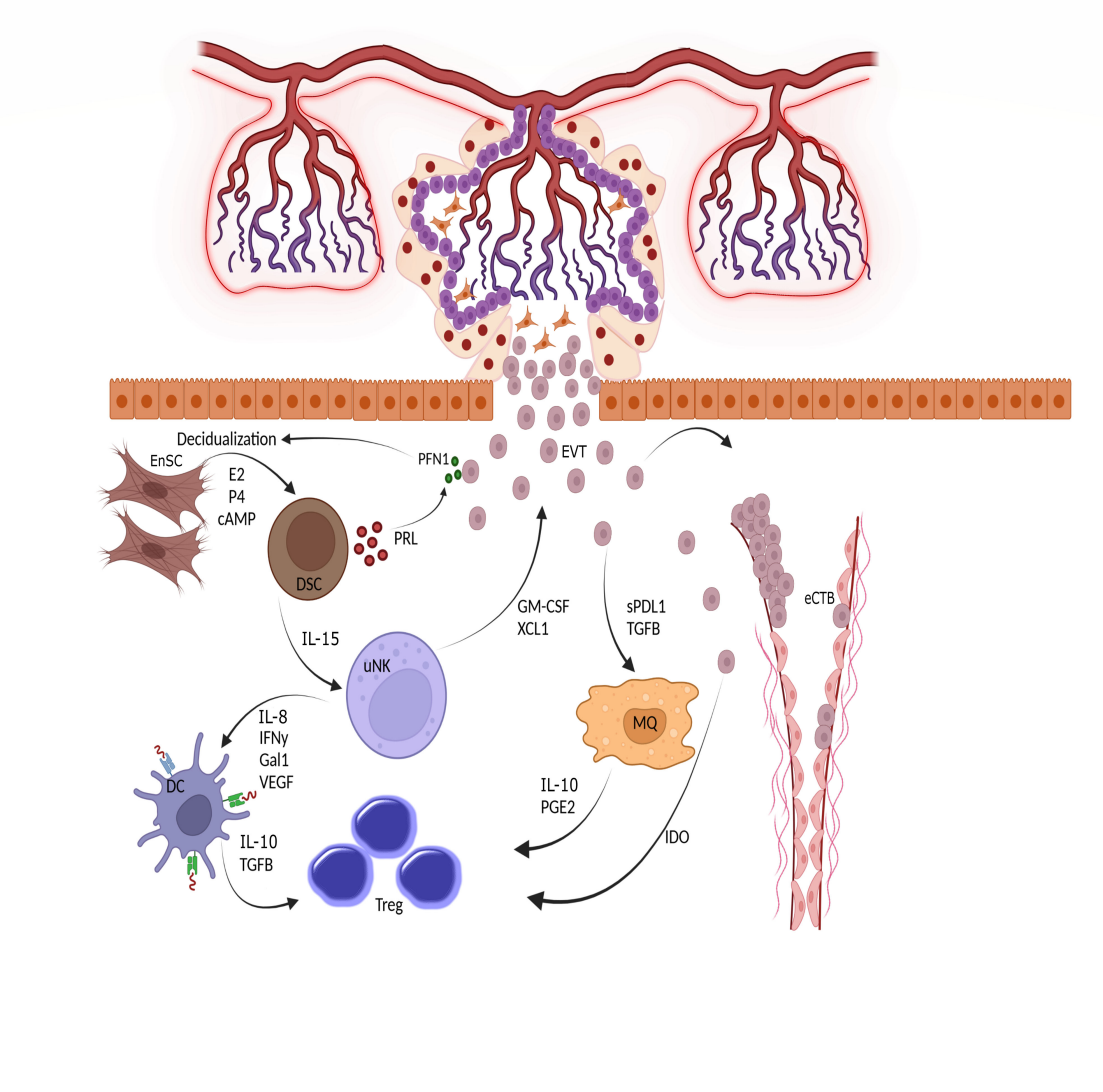
Triad interactions between trophoblast, decidual stromal cells and immune cells. Following implantation, the placental cytotrophoblasts in anchoring villi fuse together to form multinucleated syncitiotrophoblasts, which further differentiated to invasive extravillous trophoblasts (EVTs). The EVTs then invade the uterine tissues up to inner third of the myometrium. Some of the EVTs replace the endothelial cells and are called endovascular cytotrophoblasts (eCTBs). Secreted products from the invaded trophoblasts then modulate the function of endometrial stromal and immune cells. For example, Profilin-1 (PFN1) secreted from trophoblasts enhances the differentiation of endometrial stromal cells (EnSCs) into the epitheloid secretory decidual stromal cells (DSCs) in a process called decidualization through the action of estrogen (E2), progesterone (P4) and cyclic adenosine monophosphate (cAMP). Prolactin (PRL) is an important secreted product of DSCs and increases the production of PFN1 by trophoblasts. DSCs are master regulator of endometrial immune cells and help the function and survival of uterine natural killer cells (uNK), the most frequent immune cells in endometrium, by producing IL-15. The uNKs then produce factors such as IFN-γ, IL-18, Galectin-1, and VEGF to induce a tolerogenic phenotype in dendritic cells (DCs). Tolerogenic DCs are responsible for induction of regulatory T cells (Tregs) to maintain a pregnancy- friendly environment in the endometrium. These Tregs can also be induced by macrophages (MQs) under the influence of soluble products secreted from trophoblasts. Trophoblasts can directly induce Tregs using indoleamine 2,3-dioxygenase (IDO), an enzyme that degrades tryptophan. The available *in vitro* data has shown that the immune cells like uNKs can enhance the migration of trophoblasts through the secretion of chemokines. Hence, interaction between trophoblasts, EnSCs and uterine immune cells form the endometrial environment, in which each cell type modulate the function of the others. GM-CSF, Granulocyte-macrophage colony stimulating factor; XCL1, X-C motif chemokine ligand 1; IL, Interleukin; IFN, Interferon; Gal1, Galectin 1; VEGF, Vascular endothelial growth factor; TGF, Transforming growth factor; PGE2, Prostaglandin E2.

The connection between the placenta and decidua is bidirectional, with the secreted factors of each tissue regulating the function of the other tissue (**Figure 3**). For example, a recently-discovered small peptide secreted by viable embryos called pre-implantation factor (PIF) enhances decidualization of Endometrial Stromal Cells (ESCs) (36). In addition, Profilin-1 (PFN1), an actin-binding protein secreted by EVT, enhances the proliferation and decidualization of ESCs *in vitro*. HCG, produced by trophoblasts, maintains progesterone production by ovaries, which is necessary for decidualization. Reciprocally, conditioned media from DSCs increased the secretion of PFN1 by EVTs (37). Interestingly, EVs derived from endometrial stromal cells promote not only the decidualization process but also the differentiation of trophoblast stem cells into the EVT lineage. The increased expression of human leukocyte antigen (HLA)-G, matrix metalloproteinase 2 (MMP2), and integrin alpha (ITGAV) as markers of EVT differentiation validate the effect of stromal-derived EV (38). Moreover, these EVs could upregulate the expression of N-cadherin in trophoblasts, which is a critical marker of invasion (39).

Regardless of decidualization, ESCs themselves could regulate the invasion and migration of trophoblasts. Recent evidence has pointed out that the inflammatory environment generated by ESCs promotes the migration and invasive capacity of trophoblasts in a process modulated by tumor necrosis factor (TNF)-α signaling (40). The process of trophoblast invasion, as directed by DSCs, is a necessary step for blastocyst implantation. The implantation process consists of three stages: apposition, adhesion, and invasion. All phases of implantation are controlled by trophoblasts in different stages of differentiation. The apposition and adhesion phases are promoted by the stable connections between STBs and uterine epithelium. Then, with the penetration of STBs through the epithelium, the invasion phase initiates; however, successful implantation is not solely controlled by the trophoblast itself. It requires interconnections between the trophoblasts and uterine cells. These dialogs include several molecules and mediators. Among them are integrins, which are critical for the attachment of trophoblast to uterine epithelium. Like endometrial cells that express integrins on their surfaces, trophoblasts express α3, α5, β1, β3, β4, and β5 integrins, and their repertoire is modulated during invasion and differentiation of trophoblast cells (41–43). The process of trophoblast attachment to the uterine epithelium is also modulated through their interaction with extracellular matrix (ECM) components. Evidence suggests that, in addition to uterine cells, trophoblasts also produce ECM components like laminin, fibronectin, and vitronectin to increase their production of MMP, which is necessary for their invasion in an autocrine manner (44). Growth factors like epidermal growth factor (EGF) are also produced by trophoblasts to induce their invasion, differentiation, and proliferation (45). Transforming growth factor (TGF)-β, expressed by trophoblasts, was found to inhibit trophoblast invasion and proliferation through reducing MMP-9 and stimulating MMP inhibitors like tissue inhibitor of metalloproteinase (TIMP) (46). Trophoblasts also produce hCG, which increases MMP-9 and activates adenylate cyclase to produce cyclic adenosine monophosphate (cAMP) to boost their invasiveness (44). cAMP is also necessary to initiate endometrial stromal decidualization (47). In addition to the critical role of trophoblasts in implantation, the successful development of the placenta is dependent on implantation and decidualization. Taken together, decidualization, placentation, and implantation are three sides of a triangle that are necessary for maintaining a successful pregnancy.

## Bidirectional crosstalk between endometrial immune system and placenta

The coexistence of two genetically distinct individuals throughout gestation was pointed out by Peter Medawar following his studies on skin grafts. This scientific view of pregnancy, known as ‘Nature’s transplant,’ helped researchers better understand the interaction between the fetus and the maternal immune system (48). As an interface between fetal and maternal circulations, the placenta modulates the endometrial immune system. Placenta-derived factors help shape the function of immune cells in the uterus, and postnatal immune outcomes have also been associated with placental immune functions (49). Conversely, placental development and function are regulated by the local maternal immune system.

The first evidence for the regulation of placentation by the immune system comes from studies on one of the pregnancy complications known as preeclampsia (PE). Shallow trophoblast invasion in this syndrome causes maternal endothelial cell dysfunction and hypoxia in the developing fetus. Li and Wi, reported that women who have experienced preeclampsia in a previous pregnancy might exhibit protection when conceiving with a new partner. This finding could be aligned with the typical attributes of the immune system and highlight the central role of immunological specificity and memory against paternal antigens during pregnancy (50, 51).

There are two sites where fetal cells meet decidual immune cells: the large contact area between STB and maternal blood and the site of EVT-maternal decidua interaction. Endometrial immune cells have evolved mechanisms to simultaneously tolerate fetal antigens and regulate the invasion of trophoblasts (48). Bidirectional crosstalk between maternal immune cells and the placenta is exemplified by the unique functions of uNKs, the dominant leukocyte population in the first few weeks of pregnancy. Activation of Killer Immunoglobulin-like Receptor (KIR)-DS1 on uNKs by HLA-C2 on trophoblasts stimulates the production of soluble Granulocyte-Macrophage Colony Stimulating Factor (GM-CSF) and XCL1 from uNKs leading to increased migration of trophoblasts *in vitro* (52). This interaction also stimulates uNKs to secrete pro-inflammatory, angiogenic, and proteolytic mediators to help trophoblast invasion. Recent evidence has shown that extracellular matrix proteins such as fibronectin and the pro-inflammatory cytokine Interferon, (IFN)- γ, upregulate the expression of non-classical HLA molecules on trophoblasts. Interestingly, only first-trimester trophoblasts but not term trophoblasts respond to this stimulation (53). uNK cells can also orchestrate hemochorial placentation by regulating hypoxia, the key intrinsic regulator of placentation. In uNK-depleted mice, decreased oxygen tension stabilized hypoxia-inducible factor (HIF)-1 protein causing trophoblasts to become more invasive (54). Moreover, uNKs regulate the development of uterine spiral arteries by the production of VEGF (55). Mutually, the placenta also shapes the uterine immune system, and in this regard, uNKs play a crucial role. Classical HLA-C, and non-classical HLA-E and HLA-G are among the HLA molecules expressed by trophoblasts; however, trophoblasts do not express classical HLA-A and B (56). Both HLA-E and HLA-C can inhibit the cytotoxic response of NK cells to the trophoblasts. HLA-G in the soluble form induces a senescent phenotype with low cytotoxicity in NK cells (57).

It has been reported that HLA-G can be induced by leptin, a placental protein which is expressed by human trophoblasts (58). Recent evidence has shown that in addition to trophoblasts, leptin induces HLA-G expression on myocytes and inhibits contraction and differentiation of these cells. Moreover, leptin at low concentration can inhibit the production of reactive oxygen species from macrophages. This tocolytic effect of leptin can be used in prevention of preterm labor (59). Leptin level has been increased during the human pregnancy. Placenta is the second leptin-producing tissue after adipose tissue in humans during pregnancy (58). In humans, leptin is induced by beta-hCG, estradiol, and insulin and mediates immune tolerance in placenta (60–62). Although leptin has also a crucial role in rodent pregnancy, the main trigger for leptin induction in mice is unknown. In pregnant mice, adipose tissue is the main source that produces leptin and placenta is not responsible for producing this hormone; however, its receptor named OB-R or leptin receptor is expressed in placenta of both species. It is reported that mice lacking leptin receptor have fewer pups and adverse parturition outcomes (63). This implies that leptin expression with its broad immunoregulatory and functional roles has an evolutionary advantage in humans and mice as well as other primates.

Macrophages are the second most frequent immune cell population in the decidua. Modulation of macrophage polarization and function by trophoblasts is conducted via paracrine-secreted factors or cell-cell contacts. There are two subpopulations of macrophages: M1 and M2. M1 macrophages participate in extensive tissue remodeling via the secretion of matrix metalloproteinases upon trophoblast invasion, while M2 macrophages are activated by secreted factors from trophoblasts such as placental-specific glycoproteins (PSGs), M-CSF, TGF-β, IL-10, IL-34, and sHLA-G5 and support angiogenesis through the secretion of VEGF. In addition, M2 macrophages are involved in tissue repair and immune tolerance to the developing fetus (64). Recent evidence has pointed out that the phenotype of the macrophages in decidua basalis and parietalis, two anatomically different parts of the placenta, are different from each other and the secreted products of trophoblasts are responsible for this event. Macrophages of basalis layer highly express CD11c and are efficient inducers of regulatory T cells (Tregs) which secrete high levels of CXCL1, CXCL5, M-CSF, and IL-10. Whereas, macrophages of parietalis layer are motile and characterized by induced expression HLA class II for activation of T cell. It has shown that the conditioned media that collected from the culture of trophoblasts can convert the phenotype of decidua parietalis-associated macrophages into the decidua basalis phenotype (65).

Trophoblasts lack Major histocompatibility complex (MHC)-II molecules and are not able to prime maternal T cell activation; instead, decidual myeloid cells uptake EVT antigens and present them to maternal T cells in uterine draining lymph nodes (56). To avoid maternal T cell activation against fetal antigens, trophoblasts regulate T cell function by expressing indoleamine 2, 3 dioxygenases (IDO), an enzyme that inhibits T cell proliferation by depleting tryptophan (66–68). Similarly, trophoblasts control uterine T cells by Programmed cell Death Ligand (PDL)1 and PDL2 expression. This interaction is crucial for maternal tolerance during pregnancy since the blocking of PD1-PDL1 interaction in the decidua results in the expansion of T helper (Th)-1 and Th17 subtypes in favor of embryo resorption (69).

Trophoblasts also secrete TGF-β, which induces the differentiation of T cells to Treg. Moreover, placental TGF-β, IL-10, TNF-related apoptosis-inducing ligand (TRAIL) as well as galectin-1 help Tregs to expand (70). Progesterone produced by the early placenta is critical for the development of DSCs and differentiation of Tregs (71). Other examples of uterine immune stem modulation by trophoblasts are the expression of HLA-G dimers by trophoblasts which induce a tolerogenic phenotype in dendritic cells (DC) (72).

Besides trophoblasts, amniotic epithelial cells (AEC) also have a profound effect on modulation of immune cells. Human AEC (hAEC) exerted an inhibitor effect on proliferation of naive CD4^+^ T cells and production of Th1 and Th17 cytokines, while induced production of Th2 cytokines and augmented differentiation of Tregs and production of TGF-β1 and IL-10 (73, 74).

There is an increasing body of evidence suggesting a noticeable link between a pro-senescent decidual response in peri-implantation endometrium and recurrent pregnancy loss (75). Defective decidualization has been postulated to impair proper modulation of endometrial immune cells in a pregnancy-friendly manner, which could potentially lead to implantation failure or miscarriage (76). This finding shows that the endometrial stromal cells are one of the main elements of the regulation of the endometrial immune system and seriously challenges the immune system defect as the primary cause of repeated miscarriage and infertility (77–79). There is a growing body of evidence showing that mesenchymal stem cell therapy of female CBA/J mice protects fetuses from resorption in CBA/J x DBA/2 abortion model and induces Treg and Th2 type cytokines profile (80–82).Considering that highly regenerative endometrial stroma harbors multipotent endometrial stem cells with properties and phenotype similar to bone marrow and adipose tissue MSC and possibly are responsible for regenerating the endometrial stroma in each menstrual cycle (83), it would be of utmost interest to investigate whether mesenchymal stem cell therapy in CBA/J mice could potentially affect decidualization.

In the past decade, exosomes have emerged as novel mediators of intercellular communication by transfer of signaling molecules (84). All the aforementioned functions of the placenta can be mediated by exosomes. Placental exosomes can be detected in maternal blood as early as 6 weeks after conception and gradually increase until peaking at term (85). Evidence suggests that placental exosomes can be internalized by NK cells *in vitro* and *in vivo* and have diverse functions such as apoptosis induction and cytotoxicity reduction (86). These placental exosomes also educate classical phagocytic monocytes to the phenotype with impaired migratory and pro-inflammatory capacities (87). Placental exosomes can also affect apoptosis, differentiation, and cytotoxicity of maternal T cells. For instance, placental exosomes carrying NKG2D ligands such as MHC class I chain-related (MIC) and UL-16 binding protein (ULBP) as their cargo can decrease the cytotoxic action of CD8+ and γδ T cells (88).

Although, with a default conception in mind of the immunoregulatory activity of trophoblasts, most researchers have come to the conclusion that these cells exert suppressive activity on the uterine immune system, interpretation of these results should be made with caution. Pregnancy is a continuous spectrum starting from implantation and ending with parturition and involves different phases with inflammatory or anti-inflammatory natures. Many cell types and mediators from maternal origin are involved and work in concert in uterine immune modulation (89–95). In this regard, the immunoregulatory activity of trophoblasts could not tell us the whole story of endometrial immune regulation. There are three-direction interactions between the placenta, maternal immune cells, and the endometrium that shape the uterine immune fate during pregnancy and, in this regard, endometrial stromal cells are among the master regulators of uterine immune system (93).

## The story of the link between placenta development and carcinogenesis

The concept of evolutionary similarities between cancer and the placenta originates from a hypothesis proposed by Scottish embryologist John Beard. He characterized cancer as ectopic trophoblastic cells (96). While not entirely comprehensive and lacking robust evidence, this hypothesis has spurred significant research in the field. Placentation and carcinogenesis exhibit numerous shared features, broadly categorized into four levels: metabolism and cell behavior, proteins and molecular signatures, signaling pathways, and tissue microenvironment (**Table 1**) (6, 144–146). The ability to invade the surrounding stroma is a crucial characteristic shared by trophoblasts and cancer cells. This similarity has led to the hypothesis that metastasis is a negative consequence of invasive placentation, termed “antagonistic pleiotropy.” Advocates of this theory argue that cancer occurs late in a human’s lifetime, making this negative consequence tolerable. Furthermore, the advantages of invasive placentation, particularly in nourishing the fetus, outweigh its negative costs (5).

**Table 1:** Different aspects of similarity between placenta and cancer.

	Placenta		Ref		Cancer		Ref
Metabolic phenomena							
Insulin resistance (IR)	Human placental growth hormone secreted from placenta induces mild IR through increasing fatty acids that interfere with glucose entrance to the cells for fetal consumption.		(97)		Inflammation induces cachexia in cancer, which induce production of pro-inflammatory mediators. Long term exposure to these mediators leads to IR.		(98)
Warburg effect	The Warburg effect is necessary for rapid growth of the placental villus.		(99)		Cancer cells prefer to metabolize glucose anaerobically rather than aerobically, even under normoxia, which contributes to chemoresistance.		(100)
Protein signature							
Human leukocyte antigen (HLA)-G		HLA-G expressed by trophoblasts interacts with inhibitory signals on NK cells to control their cytotoxicity and its expression correlates with trophoblast invasiveness.	(101)		HLA-G expression promotes tumor progression, metastasis and is associated with poor clinical outcome.	(102)	
Placental growth factor (PlGF)		Its main action is developing angiogenesis and maintaining the blood vessels through direct action, and indirectly by VEGF.	(103)		PlGF is overexpressed in some tumors and promotes angiogenesis. Cancer patients with higher expression of PlGF have poor prognosis and lower survival.	(104)	
Human chorionic gonadotropin (hCG)		hCG is an important mediator for the endometrial implantation of trophoblasts and stimulates myometrial cell proliferation.	(105)		hCG promotes cancer cell invasion and is associated with poor prognosis in cancer patients. It induces angiogenesis and serves as a diagnostic biomarker in some types of cancer.	(106)	
Indoleamine 2,3-dioxygenase (IDO)		Increased kynurnine to tryptophan ratio or enhanced IDO activity has been reported in placenta and is associated with gestational age	(67, 107)		Enhanced IDO activity has been reported in several malignancies. It helps tumor cells to escape from the cytotoxicity of cytotoxic T cells and γσT cells.	(108, 109)	
Galectins		Galectins regulate maternal-fetal immune tolerance and promote placental angiogenesis.	(110)		Galectins are involved in cancer biology, where GAL-1 and GAL-7 are protumorigenic, while GAL-4 and Gal-8 act as a tumor suppressor.	(111)	
Insulin receptor		Placenta respond to insulin for growth.	(112)		The growth of cancer cells is effectively induced by insulin.	(113)	
Mucin 1 (MUC1)		MUC1 has been found in human trophoblasts, and its expression is increased during placental development.	(114)		MUC1 protects cancer cells from apoptosis by direct binding to the P53 regulatory domain 36.	(115)	
α5β1 integrin		The expression of integrins such as α5β1 gives the ability of migration and invasion to the trophoblast.	(116)		α5β1 expression in tumor cells promotes invasion and metastasis.	(117)	
The chemokine ligand 2 (CCL2)		Expressed by Decidual fibroblasts, macrophages and EVTs, can help recruit T cells to the maternal-fetal interface.	(118)		CCL2, secreted by cancer cells or CAFs, recruit monocytes, MDSCs, and Treg into TME.	(119)	
Transforming growth factor beta (TGF)-β		TGF-β can both enhance and decrease the invasion of trophoblasts through Smad and ERK signaling pathways, respectively.	(120)		In different stages of cancer, TGF-β can both enhance and decrease the proliferation and invasion of cancer cells	(121)	
Molecular and Epigenetic similarities							
Methylation status		Global DNA methylation level is lower in placenta compared to other organs.	(122)		Global hypomethylation status exists in cancer including hypomethylation of LINE-1 elements.	(122)	
Activation of normally silenced retrotransposons		Transposable elements that are normally silenced in somatic tissues are reactivated in placenta.	(123)		Transposable elements that are normally silenced in somatic tissues are reactivated in cancer.	(124)	
Toleration of gene mutation		The placenta can tolerate gene mutation without causing maternal harm.	(125)		Cancer cells also tolerate chromosomal aberrations but with undesired consequences to the host.	(126)	
Signaling pathways							
HIF, MAPK, PI3K/AKT, MMP, and growth factor signaling pathways like IGF-1, TGF, PDGF, FGF, PGF, VEGF, etc.		These signaling pathways involved in invasion, growth, proliferation, angiogenesis and immune evasion in trophoblasts		(127)	These signaling pathways involved in invasion, growth, proliferation, angiogenesis and immune evasion in cancer	(127)	
Tissue Microenvironment Cell behavior (Trophoblasts and cancer cells)							
Epithelial-Mesenchymal transition (EMT)		In placental development, cytotrophoblasts (CTBs) can undergo pseudo-EMT and convert to extravillous trophoblasts (EVTs).		(128)	EMT is an essential step for invasion and metastasis of different cancers.	(129)	
Cell-Cell fusion		Cell-Cell fusion in placenta may help syncitiotrophoblast (STB) to defend against oxidative stress-induced damage caused by blood flow from the uterine spiral artery		(130)	Occurs in cancer to respond to gene damage after chemotherapy or radiation	(131)	
Vasculogenic mimicry (VM)		The trophoblasts that remodel the uterine spiral artery and the vessels that form in the placenta are thought to be formed by a kind of VM.		(132)	The tumor blood supply does not depend on invasion of the vascular endothelium, and the tumor itself forms channels through the process of VM.	(133)	
Cell transformation		Decidual fibroblasts experience mesenchymal-epithelial transition (MET) during decidualization.		(134)	Cancer-associated fibroblasts developed from macrophages through a process of macrophage-myofibroblast transition (MMT).	(135)	
Tissue Microenvironment Phenomena							
Hypoxia		A moderately hypoxic environment is believed to promote CTB exit from the cell cycle, induction of EMT, and acquisition of invasive ability.		(136)	Hypoxia can induce many tumor processes, such as invasion, vasculogenesis, angiogenesis and VM, to generate a nutrient supply system.	(137)	
Immune escape: Downregulation of HLA class I expression on surface		Suppression of HLA-A and -B genes prevent the recognition of trophoblast cells by the mother’s cytotoxic T cells. This suppression is complemented by expression of HLA-C and HLA-G.		(138)	The downregulation or loss of HLA class I molecules can prevent tumor cells from being recognized by CTL.	(139)	
Immune escape: Expression of Programmed cell death ligand 1 (PD-L1)		PD-L1 is expressed on the outer surface of placental syncytiotrophoblasts, which is assumed to induce maternal immune tolerance to fetal tissue via programmed death-1 (PD-1) receptors on T cell.		(140)	Expression of PD-L1 by the tumor is a way for the tumor to evade the PD-1 positive T cells.	(141)	
Extracellular matrix (ECM) remodeling		Differentiation of fibroblasts to decidual stromal cells is associated with extensive ECM remodeling.		(142)	ECM stiffening and degradation drive cancer.	(143)	

The observation that marsupials’ placentas lack the hallmarks of mammalian invasive placentation but still develop invasive skin cancer later in life challenges the accuracy of antagonistic pleiotropy. To address this discrepancy, an alternative hypothesis called “positive pleiotropy” suggests that it is not the nature of the trophoblast itself that influences invasiveness, but rather the endometrium that controls trophoblast invasion. According to this view, animals like ruminants with less-invasive placentation have evolved to suppress trophoblast invasion, reducing the risk of malignancy (5); however, the reliability of this hypothesis is questioned by shared molecular mechanisms with other biological phenomena such as wound healing. A more detailed theory, the Evolved Level of Invasibility (ELI) theory, emphasizes the role of endometrial fibroblast cells. ELI proposes that the permissiveness or resistance of endometrial stromal fibroblasts to placental invasion in each species determines the level of malignancy and metastasis. Contrary to human embryonic stem cells (ESCs), bovine ESCs resist trophoblast invasion. Supporting this hypothesis, similar behavior is observed in skin fibroblasts of each species in response to cancer cell invasion. Tissue fibroblasts in each species, with different types of placentation, have evolved to respond to invasive cancer cells similarly to how their endometrial fibroblasts react to placental invasion (147).

As trophoblasts undergo differentiation into their lineages, they undergo an epithelial-mesenchymal transition (EMT)-like process and cell-cell fusion, akin to processes observed in cancer. Differentiation of CTB into EVT and the formation of STB from CTB involve EMT and cell-cell fusion, respectively. EMT is considered a crucial step for metastasis and invasion of tumor cells. Additionally, tumor cells respond to therapeutic intervention-induced cell damage through cell-cell fusion (148, 149).

Immune escape is another aspect of similarity between trophoblasts and tumor cells. Among the shared mechanisms of immune escape is the expression of cell surface antigens to induce immune tolerance. Proliferation, vasculogenic mimicry (VM), and angiogenesis are other aspects of similarity between placentation and carcinogenesis (144). Both tumor cells and trophoblasts are highly proliferative due to high telomerase activity, overexpression of anti-apoptotic proteins such as survivin, and activation of the insulin-like growth factor (IGF) signaling pathway (150, 151). In the VM process, tumor cells and trophoblasts form vascular structures upon invasion, providing an additional blood supply different from endothelial-dependent vasculature (132, 133). Both cell types use the same genes and signaling pathways in this process, including the overexpression of matrix glycoprotein-binding galectin-3 and Mig-7, key factors for the development of an endothelial phenotype (127). Apart from VM, the expression of key angiogenic factors such as angiopoietins, VEGF, and Placental Growth Factor (PGF), which are crucial for spiral artery remodeling in placentation and tumor cell growth, is a critical step for increasing blood supply (144).

The microenvironment of cancer and the placenta exerts profound effects on the behavior of cancer cells and trophoblasts. The decidua, as the maternal side of the placenta, affects gene expression reprogramming in trophoblasts. Similarly, the tumor microenvironment (TME) can induce epigenetic alterations in cancer cells, affecting the behavior of cancer cells, such as the promotion of cancer stemness. Immune regulation mechanisms are also shared in decidua and TME. The expression of HLA-G by both trophoblasts and tumor cells to prevent NK-mediated cytolysis, production of immunosuppressive factors to inhibit the function of cytotoxic immune cells, and the recruitment of immunosuppressive cells such as Treg and myeloid-derived suppressor cells (MDSC) are among these mechanisms. The use of the same ECM proteins and cell-secreted products and the induction of hypoxia have been observed in both environments. MMPs secreted by trophoblasts and cancer cells can shape the ECM, triggering its remodeling and facilitating invasion and the recruitment of immune cells. On the other hand, hypoxia induces EMT and invasion in CTB and VM, invasion, and angiogenesis in tumor cells (152).

Pregnancy and cancer are associated with profound systemic metabolic changes. Examples of these metabolic changes include increased blood volume and hyperglycemia to meet energy requirements during pregnancy, and abnormal metabolism in a process named cachexia, which involves the loss of skeletal muscle in cancer. In both conditions, insulin resistance (IR), defined by the inefficient function of insulin to promote glucose uptake, takes place. Inflammatory mediators, including TNF-α, IL-1, and IL-6, are key factors in the induction of IR. Nevertheless, the potential signaling pathways behind this IR remain to be further elucidated. After delivery or surgical removal of the tumor, IR gradually disappears (144).

Based on the aforementioned similarities between carcinogenesis and placentation, it could be inferred that the placenta can be considered a physiological tumor (125). In this context, the expression of placenta-specific antigens in different types of cancers has motivated many researchers to target them for cancer therapy (153–158). Moreover, biological products derived from the placenta can remarkably hinder the growth, proliferation, and progression of cancers (159, 160). For example, conditioned media from the term placenta can induce apoptosis and reduce proliferation in non-small cell lung cancer. The cellular and molecular mechanisms behind this observation involve the up-regulation of caspase 3 and suppression of the proliferation marker ki67 in tumor tissues and cell lines (161). It has also been shown that placental lysate extracts are able to suppress proliferation and induce differentiation of tumor cells *in vitro* (162). Besides *in vitro* experiments, immunization with human placenta-derived antigens has been used as an effective approach for preventive and therapeutic aims in animal cancer models (157, 163).

The idea of “immunoplacental therapy” dates back to the 1970s when a Muscovite oncoimmunologist, Valentin I. Govallo, immunized cancer patients with extracts of term placental chorionic villi. He found his therapeutic approach effective in solid tumors and published his findings in the book “Immunology of Pregnancy and Cancer.” According to Govallo’s opinion, cancer vaccination creates an immunological state similar to spontaneous abortion in pregnant women, in which immunologically foreign placental tissue would be rejected. In patients receiving placental extracts selected from various types of cancers, the overall three-, five-, and ten-year survival rates significantly increased, and metastasis was also resolved. Since Govallo’s observations, researchers have focused on using placental antigens for cancer vaccination (164).

The application of placenta-derived antigens in designing prophylactic cancer vaccines has been demonstrated in research on placental heat shock protein, glycoprotein-96 (gp96), for cancer immunotherapy. Immunization of mice with placental gp96, one week prior to challenge with melanoma cells, decreased tumor volume to 45% for about one month after the injection. In addition, higher cytotoxicity of CD8+ cells was observed against melanoma cells after immunization. Similar results were reported in gp96-immunized mice after a challenge with breast cancer cells. Comparing the tumor inhibitory effects of placental gp96 vaccine with tumor cell lysate-loaded DC-based vaccine has shown similar results in melanoma and breast cancer mouse models (163). The underlying idea of using placental antigens as effective cancer vaccines led to our recent observation that immunization with placenta-specific 1 (PLAC1), a membrane-associated protein, delays tumor onset and increases survival in a mouse model of melanoma. This immunization increased humoral and cellular immune responses, as shown by higher titers of antibodies and increased killing capacity of plac1-specific CD107a+ cytotoxic T cells (157). Using an anti-PLAC1 antibody-drug conjugate has also been shown to be effective in prostate cancer immunotherapy (158).

The anti-cancer effects of placenta-derived cells, such as hAEC, have also been the focus of much research. In a mouse model of colorectal cancer, an expanded population of systemic and splenic cytotoxic T cells and reduced tumor burden were observed after vaccination with hAEC (165). In addition to hAEC itself, secreted products derived from these cells exerted similar cytotoxic and anti-proliferative effects against cancer cell lines *in vitro*. Vaccination with hAEC-derived EVs can also induce apoptosis in cancer cells by triggering the Warburg effect and arginine consumption (160).

While the antigenic similarity between placenta and cancer could be viewed as a framework for effective targeted cancer immunotherapy, lessons from other aspects of similarity are no less important for controlling cancer cell growth. Shared cell behavior, signaling pathways, metabolic activity, and, most importantly, a common network of immunosuppressive microenvironment shed light on the future direction in the field of cancer immunotherapy. Nonetheless, the central role of the endometrium in controlling trophoblast invasion should not be ignored. According to the positive pleiotropy hypothesis, it is the endometrium and its controlling mechanisms that determine the degree of trophoblast invasion and dictate the type of placentation in eutherian mammals (5). In this context, a comprehensive view of the placenta and pregnancy decidua could better shape our understanding of the pathogenesis of cancer development and provide new clues for cancer treatment.

## Placental development and pregnancy-related diseases

The placenta plays crucial protective roles for the fetus, but its structure and function are also associated with pregnancy complications such as IR, preeclampsia, and eclampsia (166–168). Malfunctioning placenta can affect the fetus, leading to preterm birth, fetal growth issues (169), and neurodevelopmental abnormalities (170, 171). Research on fetal nutrition and its essential role in healthy fetal development (172, 173) has laid the groundwork for the concept that many fetal and adult disorders originate in the placenta. In fact, future cardiovascular disorders may be both predicted and caused by placental function (174, 175).

Insufficient placental function, as reported, can influence early growth and obesity, which are associated with an earlier age at menarche. Additionally, measurements of the fetus’s size at 35 weeks of gestation closely correspond with the rate of placental growth between 17 and 20 weeks of gestation (176). Compared to infants of normal weight, the placentas of newborns with intrauterine growth restriction have smaller diameters, higher placental coefficients, and lower weights and volumes (177). Intrahepatic cholestasis of pregnancy (ICP), a liver disease during pregnancy, typically affects women in their third trimester. ICP is linked to a higher risk of spontaneous preterm birth, fetal distress, and sudden intrauterine death, causing complications for both mother and fetus. Although the exact origin of the disease is presently unknown, research suggests that maternal bile acid levels play a role. Studies indicate that high bile acid concentrations are linked to placental apoptosis. Additionally, the placenta’s function in protecting the fetus from the negative effects of potentially harmful endogenous compounds involves mitigating the effects of bile acids on the fetus (178).

Placental disorders are often categorized into ischemic and nonischemic types. Preeclampsia, and intrauterine growth retardation (IUGR), fall under conditions where the fetus lacks sufficient blood perfusion and are thus categorized as ischemic placental disorders. Although preterm labor (PTL) was once classified outside this category (179), about one-third of preterm births are associated with changes in placental ultrastructure typically seen in ischemic disease processes (180). This finding suggests a significant overlap in the mechanisms responsible for each type of pregnancy complication (22).Critical in determining life expectancy are patterns of intrauterine development and size at delivery, impacting adult rates of morbidity and mortality as well as neonatal viability. Low birth weight, according to human epidemiological studies, is linked to an increased risk of adult-onset cardiovascular and metabolic diseases such as hypertension, coronary heart disease, obesity, and type 2 diabetes, as well as a higher incidence of reproductive and neurological disorders (170, 171, 174, 175, 181, 182). Changes in the placenta’s surface area, affecting both diffusion and transporter-mediated processes, directly influence the capacity for nutrition transfer (183). In many species, there is a positive correlation between fetal and placental weight (22, 184–187). In human IUGR cases, the placental volume is on average 92.66 cm^2^ smaller in comparison to the placenta in normal pregnancy (188).

## Placenta proteome in health and disease

### Placental proteins in normal pregnancy

Transcriptome analysis indicates that 65% of all human proteins are expressed in the placenta. Within this subset, the placenta exhibits higher expression levels for 354 of these genes, the majority of which encode secreted proteins (189).

Placental proteins are not only fundamental for pregnancy maintenance and development of the fetus, but also exerts critical function in bidirectional crosstalk with endometrial immune system and establishment of maternal tolerance during pregnancy; however, our knowledge of placental proteome is still limited and these aspects must be studied in more detail to understand the dynamics of proteins in pregnancy, fetal growth and complications during pregnancy. Indeed, this information is central for our understanding of molecular mechanisms involved in placental and gestational disorders. In this regard, proteomics technology has fundamentally advanced our knowledge of placental proteins. In this context, we recently compared normal first-trimester and full-term human placenta proteomes. A total of 120 differentially expressed proteins were identified in the first trimester versus term placenta. Among these, 20 proteins with expression fold changes higher than 2 or less than 0.5 were analyzed. Accordingly, GRP78, PDIA3, ENOA, ECH1, PRDX4, ERP29, and ECHM vs. TRFE, ALBU, K2C1, ACTG, CSH2, PRDX2, FABP5, FABP4, K2C8, MESD, K1C9, MYDGF, HBG1 had significantly higher expression in the first trimester and full-term placenta, respectively. Interestingly, we found two proteins (MESD, MYDGF) that were exclusively expressed in the first-trimester placenta. These proteins play significant roles in biological quality control, programmed cell death, hemostasis and catabolic processes, protein folding, cellular oxidant detoxification, coagulation, and retinal homeostasis. They also play essential roles in reactions to chemical stimuli and stress (190). In a comprehensive study conducted by Mushahary et al., 117 proteins in the term placenta were reported. The functions of the reported proteins were categorized into eight distinct groups, including metabolism, cell stress, cytoskeletal, transport, signal transduction, nuclear proteins, translation, cell cycle, and growth (191). In a recent study, an anatomical approach was employed to unravel the proteome profile of different sub-anatomical regions of the human placenta (192). The results showed 1081, 1086, and 1101 proteins in maternal, middle, and fetal sub-anatomical regions respectively with 374 differentially expressed proteins between sample site locations and sub-anatomical regions. Notably, proteins with the anti-senescent function were decreased in the maternal sub-anatomical region, while proteins associated with a switch from ATP to fatty acid consumption were increased in the middle and fetal sub-anatomical regions.

Apart from high throughput proteomics approaches, there are some studies with a focus on isolated placental proteins. A specific placental protein, placental protein 13 (PP13), binds to β-galactoside residues on the cell surface, cytoskeleton, and extracellular matrix, generating various responses such as blood vessel adaptation, immune responses, and influencing functions like apoptosis and molecular recognition (193). PLAC1 is the product of a paternally imprinted X-linked gene (*Plac1*) with limited normal tissue expression, which plays fundamental roles in placental function and development. It is expressed in both human and mouse placenta and is necessary for placental and embryonic development. Knockout and heterozygous placentae of the Plac1-null allele inherited from the mother weighed roughly 100% more than wildtype placentae, though the corresponding embryos weighed 7-12% less (194, 195). Retrotransposon Gag-like-1 (RTL-1), also known as Peg11, is another placental protein with a known function in placenta and fetal development. Its expression during neonatal period has to be finely regulated. Inheritance of the *Peg11/RTL1* KO allele from father causes late fetal lethality due to late fetal growth retardation, whereas maternal transmission leads to overexpression of *Peg11/Rtl1* and neonatal lethality (196, 197). This protein is preferentially expressed in the labyrinth layer of the mouse placenta by the endothelial cells. In both the *Peg11/Rtl1* paternal and maternal KO placenta, severe abnormalities of the fetal capillaries were observed. This finding is in favor of the critical role of PEG11/RTL1 protein in maintaining the integrity of the feto–maternal interface of the placenta during pregnancy (198). The placenta expresses approximately 100 placental-specific proteins. **Table 2** provides examples of placental proteins and their related biological functions.

**Table 2:** Examples of proteins expressed in different placental cell types.

Protein Name	Expressing cell	Description	Biological Process	References
PEG10 PAGE4 PEG11/RTL1	Cytotrophoblasts	Paternally expressed 10 Prostate-associated gene 4 Paternally expressed 11/Retrotransposon-like 1	Apoptosis, Differentiation, Transport Transcription regulation Maintaining placental fetal capillaries	(199, 200) (201, 202) (203)
CSH1 KISS1 GCM1 PLAC1	Syncitiotrophoblasts	Chorionic somatomammotropin hormone 1 KiSS-1 metastasis suppressor Glial cells missing transcription factor 1 Placenta-Specific 1	Stimulating lactation, fetal growth and metabolism Metastasis Suppressor Transcription regulation Placental establishment and maintenance	(204, 205) (206, 207) (208, 209) (195, 210, 211)
PAPPA2 PRG2 HLA-G	Extravillous trophoblasts	Pappalysin 2 Proteoglycan 2 Human leukocyte antigen G	Hydrolase, Metalloprotease, Protease Immunity Immune Tolerance	(212, 213) (214) (215, 216)
FCGR2B LIN28B FBN2	Placental blood vessels and stroma	Fc fragment of IgG receptor IIb Lin-28 homolog B Fibrillin 2	Transcytosis of IgG across placental endothelium RNA-mediated gene silencing Promote trophoblast invasiveness and has glucogenic action	(217) (218) (219)

### Modification of placental protein in preeclampsia

Dr. David Barker initially introduced the concept of fetal origins of adult disease (FOAD), which has garnered significant attention since its inception. The FOAD hypothesis posits that events during early development profoundly impact the risk of developing adult diseases in the future. Low birth weight, serving as a surrogate marker for poor fetal growth and nutrition, is associated with conditions such as coronary artery disease, hypertension, obesity, and IR (220–227). Epidemiological evidence now establishes a connection between an individual’s susceptibility to chronic diseases in adulthood and events during their intrauterine phase of development. Influences during this period can permanently alter the functional capacity and structure of organ systems, as well as the activity of enzyme systems and endocrine axes. These effects set the stage for a diverse array of diseases that may manifest many years later, often in response to secondary environmental stressors.

The foundation of fetal development lies in the placenta, which plays a crucial role in nutrient and oxygen delivery to the fetus. Impaired placental function or its inability to adapt may compromise fetal development (175). There is accumulating undeniable evidence in the past decade regarding the fundamental role of placental phenotype, structure, physiology, and function in a variety of postnatal diseases; however, we will specifically focus here on the modification of the placental proteome in preeclampsia, a leading cause of maternal and neonatal morbidity and mortality. This information is essential for establishing novel biomarkers for the non-invasive diagnosis of this disease.

Survivors of preeclampsia face reduced life expectancy with increased risks of diabetes, stroke, and cardiovascular diseases. Indeed, babies born from preeclamptic pregnancies are at higher risk of preterm birth, perinatal death, and postnatal diseases such as neurodevelopmental disability, and cardiovascular and metabolic diseases later in life. Preeclampsia is a syndrome driven by placental dysfunction causing maternal endothelial dysfunction and systemic inflammation (228).

Only a limited number of studies have delved into alterations in placental tissue proteins related to pregnancy-related disorders. Among a total of unique placental proteins reported by Mushahary et al. (191), twenty-five proteins (mainly cell stress proteins) have been associated with PE (229–235), early pregnancy loss [ubiquitin-conjugating enzyme E2 N, calgizzarin (S100 calcium-binding protein A11), galectin-1 (236), PTL (cytoskeletal proteins) (237) and assisted reproductive technology [fascin] (238).

In a systematic review, twelve studies employing mass spectrometry-based techniques were examined, comparing samples from preeclamptic and normotensive pregnancies. Across all studies, 401 proteins with significantly altered expression in PE were identified. Comparison between studies identified 52 proteins as significant in two or more studies, which were then enriched for 22 pathways, including those previously implicated in PE such as hemostasis, immune response, and lipid metabolism. In particular, the proteins complement component 4 and apolipoprotein E displayed abnormal expression at week 12 before the clinical diagnosis of PE, suggesting their value as clinical biomarkers (239). Downregulation of antioxidant proteins (peroxiredoxin two and peroxiredoxin 3) and altered expression of stress response proteins (Hsc) 70, Heat shock protein (Hsp) gp96, and protein disulfide isomerase) has also proposed in the pathogenesis of PE (235). To assess the effect of elevated levels of neurokinin B (NKB), a tachykinin linked to systemic symptoms of PE, on the placental proteome, the proteome profile of cultured human term CTB was assessed in response to NKB treatment. The results revealed a statistically significant decrease in 20 proteins, including those crucial in antioxidant defenses (240). **Table 3** summarized alterations of placental proteins in women with PE.

**Table 3:** Alterations in the placental protein expression in PE.

Name	Description	Expression Change	References
GAPDH ANXA4 CLIC3 PRDX2 HSPB1 ALB PLG HBZ FGB FGG CAT CYP11A1 ANXA6 HSPA5 ATIC ACTG1 PAPPA2 FLT1 FTH1	Glyceraldehyde-3-phosphate dehydrogenase Annexin A4 Chloride intracellular channel protein 3 Peroxiredoxin-2 Heat shock protein beta-1 Albumin Plasminogen Hemoglobin subunit zeta Fibrinogen beta chain Fibrinogen gamma chain Catalase Cholesterol side-chain cleavage enzyme, mitochondrial Annexin A6 Endoplasmic reticulum chaperone BiP Bifunctional purine biosynthesis protein ATIC Actin, cytoplasmic 2 Pappalysin-2 Vascular endothelial growth factor receptor 1 Ferritin Heavy chain	Up Up Up Up Up Down Up Up Down Down Up Up Up Up Up Down Up Up Down	(241–243) (243–245) (235, 241, 246–248) (247, 249–251) (242–244, 246, 247) (241, 244, 247–249) (241, 249, 252) (241, 248, 250) (244, 249, 250) (248–250) (242, 249–251) (241, 248, 253) (241, 249, 252) (243, 246, 247, 249) (241, 248, 251) (235, 244, 254) (241, 245, 248) (245, 248, 249) (255, 256)

### Placental proteins as biomarkers for early diagnosis of Preeclampsia

There is a necessity to identify and establish gestational biomarkers that can be employed for monitoring pregnancies, and predicting adverse outcomes. While utilizing proteomic biomarkers in placental tissue is one approach for assessing health, nutritional status, and disease, it is practically challenging for the early diagnosis of gestational diseases. Therefore, recent trends primarily focus on the non-invasive examination of placental proteins in peripheral blood. Human placenta harbors numerous proteins, most of which are secreted to the maternal circulation during pregnancy. Notable examples of these secreted proteins include human chorionic gonadotropin, human placental lactogen (hPL), human GH variant or placental growth hormone, leptin, corticotropin-releasing hormone, placental growth factor (PlGF) all of which are secreted by the placenta into the mother’s bloodstream (31, 58, 178, 257–265).

The composition of maternal plasma proteome is steadily altered throughout gestation, which is in part due to the changes in the placental proteins secreted to the maternal circulation. This has offered a novel perspective for the precise timing of placental protein expression relative to gestational age, the “placental proteomic clock”. Keeping this concept in mind, several researchers have unveiled changes in the placental proteome during different gestational periods. In a cohort study, Degnet et al. identified five placenta-derived proteins capable of predicting conceptional age, including chorionic somatomammotropin (CSH1/2), biglycan (BGN), glypican 3 (GPC3), inter-alpha-trypsin inhibitor heavy chain H5 (ITIH5), and lysosomal alpha-glucosidase (GAA) (266). In this study, Degnes et.al., among nearly 5000 measured proteins in maternal circulation, described 256 placenta-derived proteins and 101 proteins absorbed by the placenta. Of these, 101 placenta-derived proteins were deemed placenta-specific, forming two clusters with distinct developmental patterns throughout gestation. This innovative concept may serve to monitor deviations in the developmental patterns of placenta-derived proteins across gestation, indicating potential placental dysfunction.

PlGF, a member of the VEGF family predominantly expressed in the placenta, is crucial for normal pregnancy. PlGF concentrations are initially low in the first trimester of an uncomplicated pregnancy, increasing from week 11 to 12 and peaking at week 30, followed by a decline. Decreased PlGF expression in the placenta is associated with disruptions in placental development, maturation, and the onset of conditions like PE. Since PlGF’s primary role outside the placenta is angiogenesis in response to pathological ischemia or injury, a low level of PlGF serves as an indicator of abnormal placentation. This is supported by observations that women without preeclampsia who give birth to small for gestational age babies also exhibit low PlGF levels early in pregnancy (267). Similarly, serum levels of inhibin A, a glycoprotein of human STB, have been explored for predicting and evaluating the severity of PE. The deficient trophoblastic invasion in PE leads to ischemic damage to the STB’s surface layer. This modification is suggested to contribute to the increased release of inhibin A into the maternal circulation. In comparison to the group without adverse pregnancy outcomes, elevated serum levels of inhibin A during the second trimester not only pose a risk for preeclampsia but also for gestational diabetes mellitus, preterm delivery, and low birth weight (268, 269).

PP13 has emerged as a biomarker for predicting preeclampsia. Lower levels of maternal serum PP13 in the first trimester are associated with the development of PE and intrauterine growth restriction (193, 270, 271).

Measurement of maternal serum levels of PlGF and Pregnancy-associated Plasma Protein-A (PAPP-A), combined with maternal risk factors (e.g., obesity, hypertension, maternal age, etc.) and uterine artery measures by Doppler ultrasound, is proposed to have sensitivity of about 95% for detection of early-onset PE (diagnosed < 34 weeks) (272). In addition to alterations in placental protein expression, there are reports indicating that placental stress can lead to maternal physiological maladaptation to pregnancy by causing abnormal glycosylation of placental-derived factors. Endoplasmic reticulum (ER) stress leads to diminished complexity and sialylation of trophoblast protein N-glycosylation, and aberrant glycosylation of VEGF leading to its reduced bioactivity. ER stress affects the expression of 66 out of 146 genes annotated with ‘protein glycosylation’ and diminishes the expression of sialyltransferases (273). **Table 4** summarizes the serum proteins associated with early onset PE.

**Table 4:** Serum proteins associated with early onset PE.

Name	Description	Expression change	Reference
HP	Haptoglobin	Down	(274–276)
IGKV1-9	Immunoglobulin kappa variable cluster	Down	(275, 276)
CST3	Cystatin C	Up	(276, 277)
ITIH3	Inter alpha trypsin inhibitor heavy chain 3	Up	(275, 276)
FN1	Fibronectin 1	Up	(275, 276)
C4B	Complement protein	Down	(275, 277)

Of note, most of the evaluated markers have been found when the diagnosis of PE has been already established. Despite the identification of proteins that are differentially expressed in patients with PE, few studies have been conducted to find a specific and reliable marker for early prediction of the disease onset. Using a machine-learning approach, a recent study combined multi-omics datasets (transcriptome, proteome, etc.) of plasma, urine and vaginal swab, collected from pregnant women during the first 16 weeks of pregnancy to find a panel of markers for early diagnosis of PE. Plasma proteome and urine metabolome were the most accurate predictive models for PE and, in each model, top proteins and metabolites were identified. Adenine, isovalerylglutamic acid, uric acid ribonucleoside, 1,5-anhydroglucitol, dehydroepiandrosterone, sialyllactose, Nϵ-acetyl-L-lysine, and nonanoylcarnitine were the top urine metabolites that predict PE with high accuracy. Besides, serum proteins leptin (LEP), VEGF, L-selectin (SELL), E-selectin (SELE), IL-24, IL-22, tyrosine-protein kinase transmembrane receptor (ROR1), C-X-C motif chemokine ligand 10 (CXCL10), and SPARC-like 1 (SPARCL1) were found to have the top predictive value for PE. Along with the plasma proteome and urine metabolome, the immune cell dynamics of the single cells isolated from the patient’s blood between the first and second trimesters can also predict the onset of PE and correlated with the aforementioned markers in plasma and urine. Basal pSTAT1 (phosphorylated signal transducer and activator of transcription) and STAT5 signaling as well as basal NF-κB (Nuclear factor kappa B) levels in immune cells were the immune features that correlated with top predictive plasma proteins. There was also significant correlation between the top predictive proteins and metabolites with clinical data of the patients. Upon final univariate analysis of all the predictive features, the LEP, CCL23, and FAM3D had significant association with PE outcome (278).

## Placental proteins as biomarkers for cancer diagnosis and target discovery

**Figure 4:**
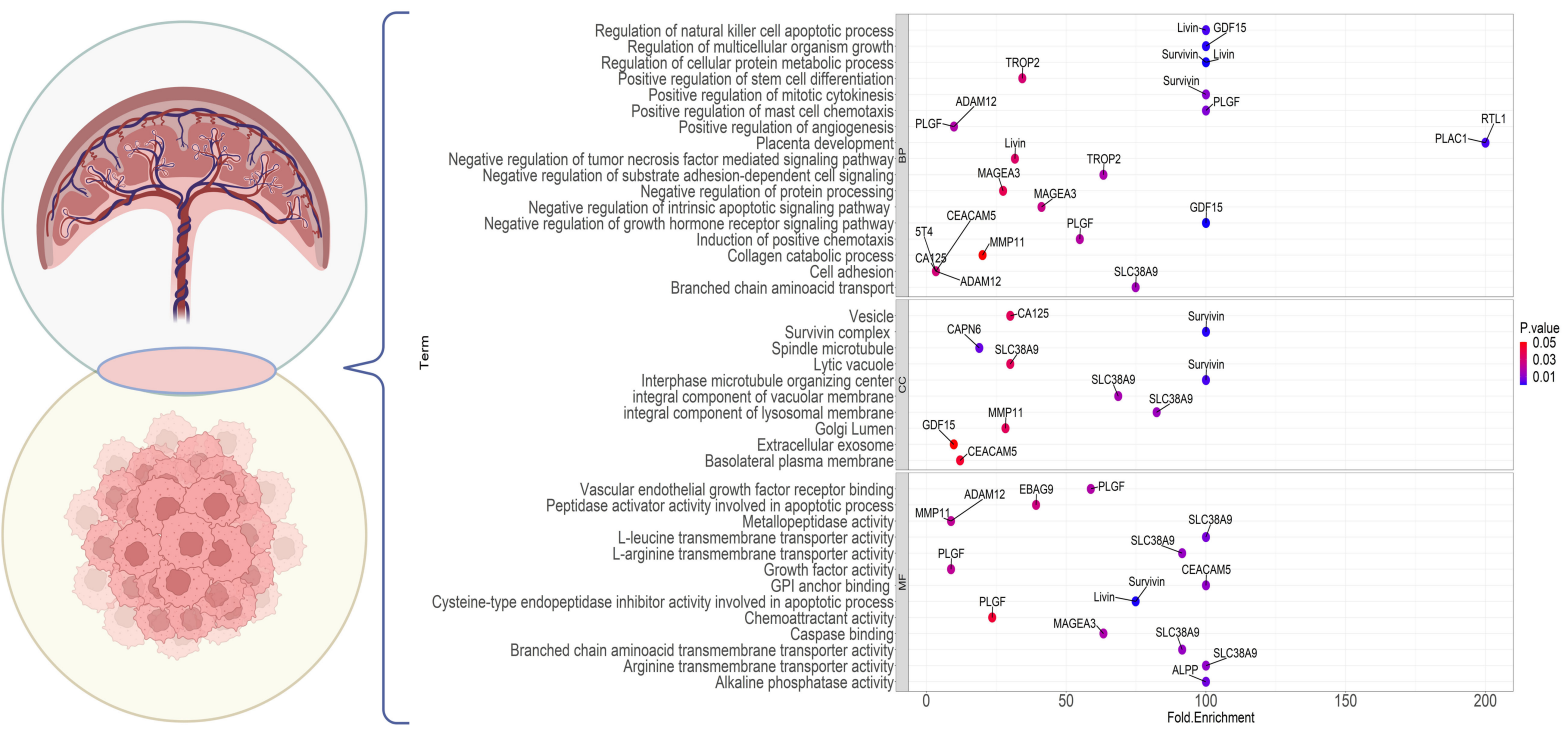
Gene Ontology enrichment analysis of the genes commonly expressed in cancer and placenta. The dot plot shows the top enrichment terms for the markers with a shared expression between the placenta and tumor cells. The color of each dot indicates the enrichment P value and the x-axis shows the enrichment fold. Each part points to one of the GO sections; BP, Biological Processes; CC, Cellular Component; MF, Molecular Function.

Based on investigations into differentially expressed proteins in cancer, certain proteins have been identified with high expression in the testis. Coined as “cancer-testes antigens” (CTAs) by Old and Chen in 1997 (279, 280), these proteins form over 70 families with more than 170 members, extensively documented in various cancer databases (281). Intriguingly, some of these proteins also exhibit elevated expression in placental tissues, leading to the emergence of a new term, “cancer placental antigens” (CPAs) or more comprehensively, “cancer testes placental antigens” (CTPAs). **Figure 4** illustrates several CPAs, their cellular compartmentalization, and enrichment in diverse biological pathways. Certain markers, like cancer antigen 125 (CA-125) and carcinoembryonic antigen cellular adhesion molecule-5 (CEACAM-5), already serve as serum tumor markers for pancreatic and ovarian cancers (282, 283); however, their limited specificity and sensitivity hinder their reliability for accurate diagnoses (155). Notably, Melanoma-associated antigen 3 (MAGE-A3), a CTPA, has proven to be an attractive target in undifferentiated sarcoma (284).

PlGF, utilized as a biomarker for predicting and diagnosing PE, has demonstrated overexpression in cancer and its associated stromal cells (104). Trophoblast surface glycoproteins TROP2 and 5T4 hold prognostic significance in at least 13 different types of cancers (285, 286). Antibodies against survivin, livin, and PLAC1 are detected in sera across various cancer types (287–290). Recent mining of transcriptomic databases has identified MMP11 and ADAM metallopeptidase domain 12 (ADAM12) as potential CPAs, with MMP11 exhibiting high transcript levels in 25 different types of cancers (155). PLAC1, an X-linked trophoblast antigen, is ectopically expressed in a large set of cancer cell lines and several cancers, including melanoma, breast, cervical, prostate, ovary, liver, and colorectal cancers (156, 158, 195, 291, 292). More recently, RTL1 has been introduced as a placenta-specific protein with ectopic expression in cancer, particularly evident in hepatocellular carcinoma (HCC) (293), melanoma (294), and breast cancer (295). In melanoma, RTL1 promotes cell proliferation through the Wnt/β-Catenin signaling pathway (294). Preliminary observations indicate high RTL1 expression in breast cancer cell lines and tissues, correlating significantly with histological grade and vascular invasion (295). Further research is required to uncover the cancer-modulatory effects of RTL1 *in vivo*.

Growth/differentiation factor-15 (GDF-15) is a placental protein, which is barely detected in most somatic tissues in humans. It is a member of the TGF-β superfamily and is highly expressed across various cancer types (296, 297). GDF-15 is induced under stress conditions to maintain cell and tissue homeostasis. Reportedly, high blood levels of GDF-15 are associated with inflammatory conditions, which is the hallmark of myocardial ischemia, and especially cancer (297).

Numerous studies have concentrated on tumor-associated autoantibodies as potential cancer biomarkers (298–301). Considering common antigens expressed in both placenta and various cancers (CPAs), autoantibodies against placenta proteins could be viewed as potential serologic cancer biomarkers. We recently reported that patients with breast, gastric, and colorectal cancers produce detectable levels of placenta-reactive autoantibodies. This was extremely the case for breast cancer, in which sera from early stage breast cancer patients contained antibodies reactive with the first-trimester placenta and reacted with a scattered subpopulation of cells, probably trophoblast stem cells (153).This approach, could be utilized for non-invasive cancer screening at early stages, paving the way for new strategies in targeted immunotherapy for cancer patients. Profiling of autoantigens by protein array technology for discovery and point-of-care type investigations are a promising approach for prognostics and diagnostics (302).

## Concluding remarks

Despite the undeniable importance of the placenta in eutherian pregnancy, this vital organ has not been studied as much as it deserves. New findings show that the placenta, in addition to being the major anatomical site to provide nutrients to the developing fetus, has an undeniable role in regulating the microenvironment of the endometrium and preparing it for a successful pregnancy. Despite the conventional belief of the main role of maternal factors, placental cells, and its secretory factors exert a central role in regulating the maternal immune system and inducing immunological tolerance of pregnancy. Placental proteome and circulating levels of placental proteins are reliable sources for investigating and identifying gestational diseases. Interestingly, there is a very fascinating connection between placentation and carcinogenesis. Accordingly, the type of placentation in eutherian mammals and adaptation of endometrium to placental invasion are determining factors in the prevalence of the type of invasive cancers. In this context, a deep knowledge about the placenta and pregnancy decidua could better shape our understanding of the pathogenesis of cancer development and provide new clues for cancer treatment. In spite of being the star of the evolution of mammals, yet placenta is generally seen as a waste organ! It is not the placenta’s fault that it has languished in such obscurity. No one has bothered to speak up on its behalf.

## Author contributions

SK-S: Writing – original draft, Writing – review & editing. NV: Software, Writing – review & editing. SS: Conceptualization, Data curation, Writing – review & editing. KZ: Writing – original draft, Formal analysis, Methodology. AS: Data curation, Formal analysis, Investigation, Writing – review & editing. MJ-T: Project administration, Supervision, Writing – review & editing. A-HZ: Project administration, Supervision, Validation, Writing – original draft, Writing – review & editing.
